# The anxiolytic-like effects of ginsenoside Rg3 on chronic unpredictable stress in rats

**DOI:** 10.1038/s41598-018-26146-5

**Published:** 2018-05-17

**Authors:** Jia-ning Xu, Li-fang Chen, Jun Su, Zhi-li Liu, Jie Chen, Qing-fen Lin, Wei-dong Mao, Dong Shen

**Affiliations:** 1 Department of Emergency, The Affiliated Jiangyin Hospital of Southeast University Medical College, Jiangyin, Jiangsu 214400 P.R. China; 2 Department of Oncology, The Affiliated Jiangyin Hospital of Southeast University Medical College, Jiangyin, Jiangsu 214400 P.R. China

## Abstract

The present study is to evaluate the anxiolytic-like activities underlying ginsenoside Rg3 (GRg3). The anxiolytic-like activities were induced by GRg3 (20 and 40 mg/kg, i.g), evidenced by blocking the decreased time and entries in the open arms in elevated plus maze test and by reversing the increased latency to feed in novelty-suppressed feeding test. In addition, the decreased levels on progesterone, allopregnanolone, serotonin (5-HT) in the prefrontal cortex and hippocampus of chronic unpredictable stress (CUS) were blocked by GRg3 (20 and 40 mg/kg, i.g). Furthermore, the increased corticotropin releasing hormone, corticosterone and adrenocorticotropic hormone were blocked by GRg3 (20 and 40 mg/kg, i.g). Collectively, the anxiolytic-like effects produced by GRg3 were associated with the normalization of neurosteroids biosynthesis, serotonergic system as well as HPA axis dysfunction.

## Introduction

Anxiety disorder is one of the serious mental diseases^1^. The symptoms of anxiety generate in the neuropsychiatries, including panic, generalized anxiety, post traumatic stress disorder (PTSD) *et al*.^2,3^. The involved factors are remain uncleared, although considerable attentions have been focused on this disorder.

The dysfunction of monoaminergic neurotransmission is an important factor underlying the pathology of anxiety^4,5^. The monoaminergic hypothesis indicates monoamines in the brain (e.g prefrontal cortex and hippocampus) are associated with the etiology of anxiety^5,6^. Most of the anxiolytic-like effects of drugs are associated with the monoaminergic activities, such as the inhibited reuptake on serotonin (5-HT) and other monoaminergic metabolites. Following the anxiolytic treatments, the elevated levels on monoamine neurotransmitters were compared with that of controls in the brain^7^.

A number of drugs are considered as the usual treatments for anxiety^5^, such as selective serotonin reuptake inhibitors (SSRIs) as well as selective serotonin and noradrenaline reuptake inhibitors (SNRIs)^1,8^. However, multiple side effects could be induced by SSRIs and SNRIs, i.e cognitive deficits, dependence, sedation, withdrawals, *et al*.^9,10^. Thus, more efforts are essential to search for the novel anxiolyic agents.

**Figure 1:**
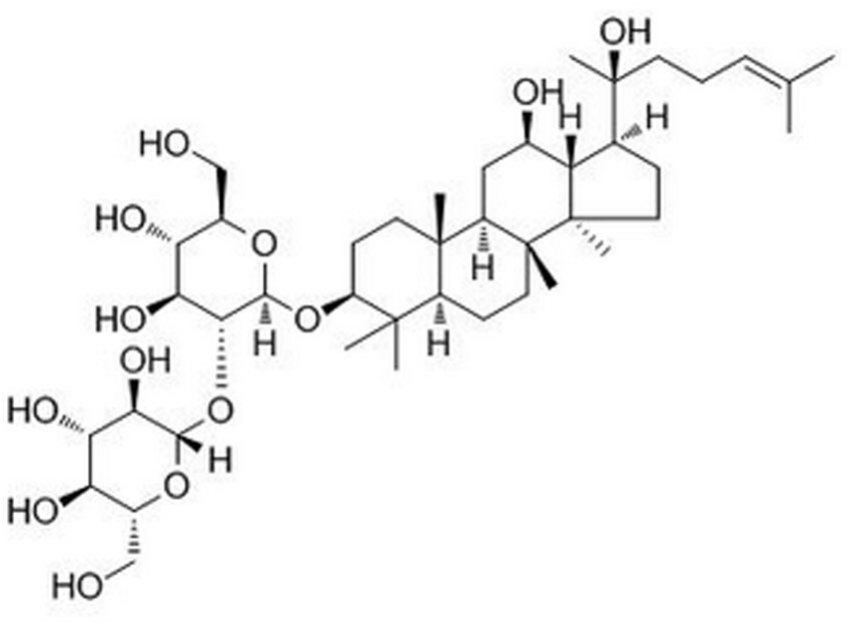
The chemical structure of ginsenoside Rg3 (GRg3).

More attention has been paid for the plant preparations and natural extracts to combat the anxiety disorders^5,11^. Ginsenoside Rg3 (GRg3), a protopanaxatriol-type compound, is one of the active components in the stem leaves and root of ginseng (Fig. 1)^12^. Various pharmacological effects could be produced by GRg3, such as antioxidant, anticancer, anti-inflammatory, anti-aging, *et al*.^12–15^. Besides, the potential effects on attenuating memory impairments, neurotoxicity, depressive-like behavioral deficits could also be elicited by GRg3^16,17^. However, its anxiolytic-like effects are still not fully known.

Beside the abnormalization of monoaminergic function, the decreased levels on neurosteroids (e.g progsterone and allopregnanolone) are also correlated with anxiety^5,18^. For instance, the decreased levels on neuroactive steroids (particularly allopregnanolone) in the cebrospinal fluid and blood may induce anxiety, depression, PTSD, impulsive aggression, *et al*.^19^. In the contrary, normalizing the decreased neurosteroids may be considered as one of the promising pharmacological strategies to defend anxiety.

More studies on the factors involved anxiety, like disdurbance of hypothalamic-pituitary-adrenal (HPA) axis, may provide the new perspectives on the pathology and the potential identification for therapeutic targets to ameliorate the anxiogenic-like behavioral deficits. HPA axis, consists of a feedback loop that including the hypothalamus, pituitary as well as adrenal glands. The dysregulation of HPA axis that maybe one of the possble factors to anxiety, which is considered to be induced by chronic stress^19,20^. The hyperactivity of the HPA axis in stress/anxiogenic-like behavioral deficits is thought to be particularly involved in reduced feedback inhibition via the endogenous hormones, i.e adrenocorticotropic hormone (ACTH), corticosterone (Cort) and corticotropin releasing hormone (CRH)^21–23^.

The animal model of chronic unpredictable stress (CUS), a classical evaluation for anxiogenic-like behavioral deficits^20^, is prepared to assess the anxiolytic-like effects of GRg3. To further investigate the involved molecular factors, the biosynthesis of neurosteroids, HPA axis activation as well as the levels on monoamines were also observed.

## Materials and Methods

### Animals

The rats (Sprague-Dawley, 180–200 g) were maintained in a 12h- light/dark cycle, humidity (45–55%)- and temperature (22–24 °C)- controlled condition with food and water available freely. Total number of animals was sixty that were divided into six groups and ten in each group. The study was conducted according to the National Institute of Health Guide for the Care and Use of Laboratory Animals which was approved by institution of Academy of Military Medical Sciences.

### Preparation of the *chronically* unpredictable stressed animal model

The model was prepared based on the previous study^24^ and shown in Table 1. Except for controls, the rats were exposed to the administrations randomly and continuously as below: (1) white noise (approx. 120 dB), (2) forced swimming (5 min at 8–10 °C), (3) food or water deprivation for 24 h, (4) tail pinch for 180 s, (5) soiled cage (150 mL water in 80 g sawdust bedding), (6) 45° cage tilt, (7) overnight illumination, (8) restraint for 2 h, and (9) stroboscopic illumination (90 flashes/min).

**Table 1:** Chronic unpredictable stress schedule.

Groups	Condition			
	Week 1	Week 2	Week 3	Week 4
Monday	Overnight stroboscopic: 12 h	Force swimming: 5 min	White noise: 1 h	Food derivation: 24 h
Tuesday	Water deprivation: 24 h	Water deprivation: 24 h	Force swimming: 5 min	Tail pinch: 1 min
Wednesday	Tail pinch: 1 min	White noise: 1 h	Overnight illumination: 12 h	Overnight illumination: 12 h
Thursday	Force swimming: 5 min	Restraint: 2 h	Water deprivation: 24 h	Restraint: 2 h
Friday	White noise: 1 h	Food derivation: 24 h	Tail pinch: 1 min	White noise: 1 h
Saturday	Restraint: 2 h	Overnight stroboscopic: 12 h	Soiled cage: 24 h	Soiled cage: 24 h

### Drugs

Both GRg3 and sertraline were obtained from Sigma-Aldrich (USA), dissolved in Dmethyl sulfoxide (DMSO, <0.1%) and prepared in physiological saline. The doses of GRg3 (10, 20 and 40 mg/kg i.g) were selected according to its antidepressant-like effects^15^. Sertraline (15 mg/kg i.g) was prepared as a positive control in the behavioral assessments that based on the previous study^24^.

### Behavioral paradigms and drugs treatments

**Figure 2:**
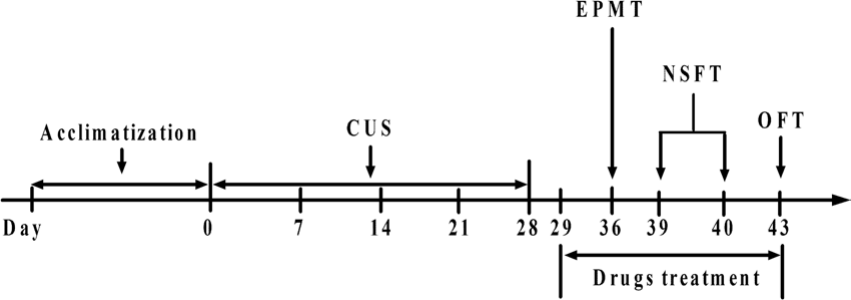
Treatment and behavioral test schedules. Animals were subjected to CUS from day 1 to 28. From day 36 through 43, animals were performed various behavioral tests that were composed of various behavioral tests: elevated plus maze test (EPMT) (on day 36), novelty-suppressed feeding test (NSFT) (from day 39 to 40), and open field test (OFT) (on day 43). GRg3 (10, 20 and 40 mg/kg, i.g.) and sertraline (at a dose 15 mg/kg, i.g.) were administered once daily from day 29 through 43. The drugs were administered 1 h before testing, respectively.

The animals were exposed to CUS from day 1 to 28 after the acclimatization (1 week). Each one was subject to various behavioral tests from day 36 to 43: elevated plus maze test (EPMT) (on day 36), novelty-suppressed feeding test (NSFT) (from day 39 to 40), and open field test (OFT) (on day 43). Both GRg3 and sertraline were administered by intragastric gavage (i.g.) once daily from day 29 to 43. Control animals were received by 0.9% physiological saline. When behavioral tests were performed on the days (day 36, 39, 40 and 43), the drugs were administered 1 h before the behavioral tests (Fig. 2).

Following the completion of behavioral assessment, the rats were decapitated in 24 h. The samples were collected for further evaluations, including the blood for levels on Cort, CRH and ACTH measurement as well as the brain tissues for levels on neurosteroids and monoamines quantification.

### EPMT

EPMT is a usual assessment for evaluating the anxiolytic-like effects^5^. The apparatus is 50 cm above the ground including: two closed arms with dark walls (60 × 12 × 40 cm) and two open arms (60 × 12 cm). The arms are connected by the central platform (12 × 12 cm). Each one was placed in the platform facing one of the closed arms and defined as entering an arm when four paws crossed the dividing line. Time and entries into the open arms were considered as the aniolytic indices by observers who were blind to the treatments/grouping.

### NSFT

The NSFT is another reliable and sensitive assessment for evaluating anxiogenic-like behavioral deficits^25^. After fasting for 24 h, each one was placed in the corner of the plastic box (76 × 76 × 46 cm) with a few pallets in the center. The latency was recorded within 5 min when the rat began eating (defined as biting or chewing the pallets). Moreover, the home-cage food consumption was recorded in 5 min to evaluate the effects of drugs on the feeding drive.

### OFT

The OFT was performed to evaluate whether the anxiolytic-like effects were produced by GRg3 except affecting locomotor activity^26^. The individual was placed in the corner of a plastic box (dimensions: 76 × 76 × 46 cm) which the base was divided into 16 equal squares. The crossings (all the paws placed into a new square), rears (both front paws raised from the floor), as well as fecal pallets were recorded in 5 min.

### Levels of Cort, CRH and ACTH measurement

The blood was collected after OFT in 24 h. The samples were centrifuged (4000 g, 4 °C, 30 min) and stored (−80 °C). The levels on Cort, CRH and ACTH in serum were quantified by the enzyme linked immunosorbent assay (ELISA) kits. The conjugate and sample/standard were injected to each well. Then, the plate was incubated at room temperature for 1 h. The optical density values were recorded by ELISA plate reader at 450 nm until the washes and proper color development.

### Levels of neurosteroids measurement

The dysfunction of neurosteroids biosynthesis (like progesterone and allopregnanolone) in the brain is also considered as one of the factors to anxiogenic neuropathology^5,19^. The prefrontal cortex and hippocampus were dissected after OFT in 24 h. The brain tissues were extracted and homogenized by the buffer. The tissue homogenate solutions were centrifuged (12,000 g, 25 min, 4 °C). Then, supernatants were collected. The levels of neurosteriods (e.g progesterone and allopregnanolone) were quantified by Enzyme Immunoassay kit. The optical density values were recorded by the ELISA plate reader at 450 nm.

### High-performance liquid chromatography with electrochemical detection (HPLC-ECD)

To further evaluate involved factors to the anxiolytic-like effect of GRg3, the levels on monoamine neurotransmitters were quantified by HPLC-ECD^27^. The animals were decapitated after OFT in 24 h. The prefrontal cortex and hippocampus were dissected on the ice, homogenized in the tissue lysis buffer and centrifuged (12,000 g, 20 min, 4 °C). Following that, the supernatants were filtered through a 0.45 μm pore membrane. The sample/standard solutions were injected into the reversed-phase C_18_ column. The monoamine neurotransmitters, i.e 5-HT, 5-hydroxyindoleacetic Acid (5-HIAA), dihydroxy-phenyl acetic acid (DOPAC), AD (adrenalin), DA (dopamine), HVA (homovanillic acid) and NE (norepinephrine) were quantified in the isocratic elution mode at a column temperature of 16 °C.

### Statistical analysis

The results were analyzed by GraphPad Prism 5.0 and presented as the mean ± S.E.M. Statistical significance was indicated by one-way analysis of variance (ANOVA) followed by Bonferroni’s multiple comparison tests. Differences at an alpha value (*p* < 0.05) were defined as statistically significant.

## Results

### The anxiolytic-like effects were produced by GRg3 on EPMT

**Figure 3:**
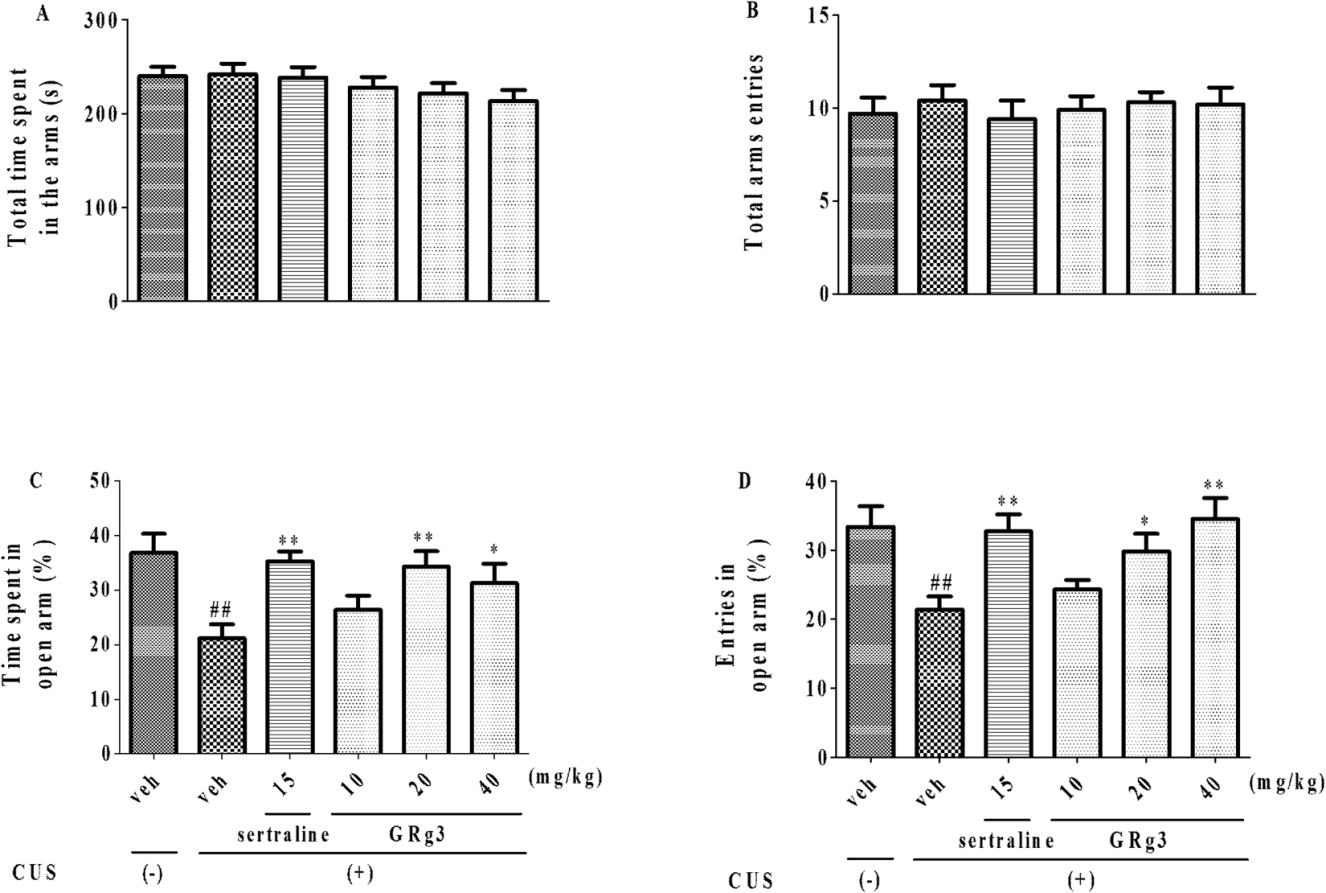
The anxiolytic-like effects of GRg3 in EPMT following exposure to CUS. The behavior was presented by percentages of time spent (**C**) in and entries (**D**) into open arms, as well as total time (**A**) and entries (**B**) in the arms. ^##^*p* < 0.01 vs. vehicle-treated CUS (−) group; *p < 0.05, **p < 0.01 vs. vehicle treated CUS (+) group (n = 10).

As observed in Fig. 3, the percentage of time (F_5,54_ = 4.382, *p* < 0.05, 3 C) and entries (F_5,54_ = 4.694, *p* < 0.05, 3D) into open arms was decreased after the exposure to CUS. However, similar to the effects of sertraline (15 mg/kg, i.g.), both decreased time and entries were blocked by GRg3 (20 and 40 mg/kg, i.g.) except affecting the total time (F_5,54_ = 1.068, *p* > 0.05, 3 A) and entries (F_5,54_ = 0.2187, *p* > 0.05, 3B) in all the arms. The results indicated that anxiogenic-like behavioral deficits could be ameliorated by GRg3 via EPMT.

### The anxiolytic-like effects were produced by GRg3 in NSFT

**Figure 4:**
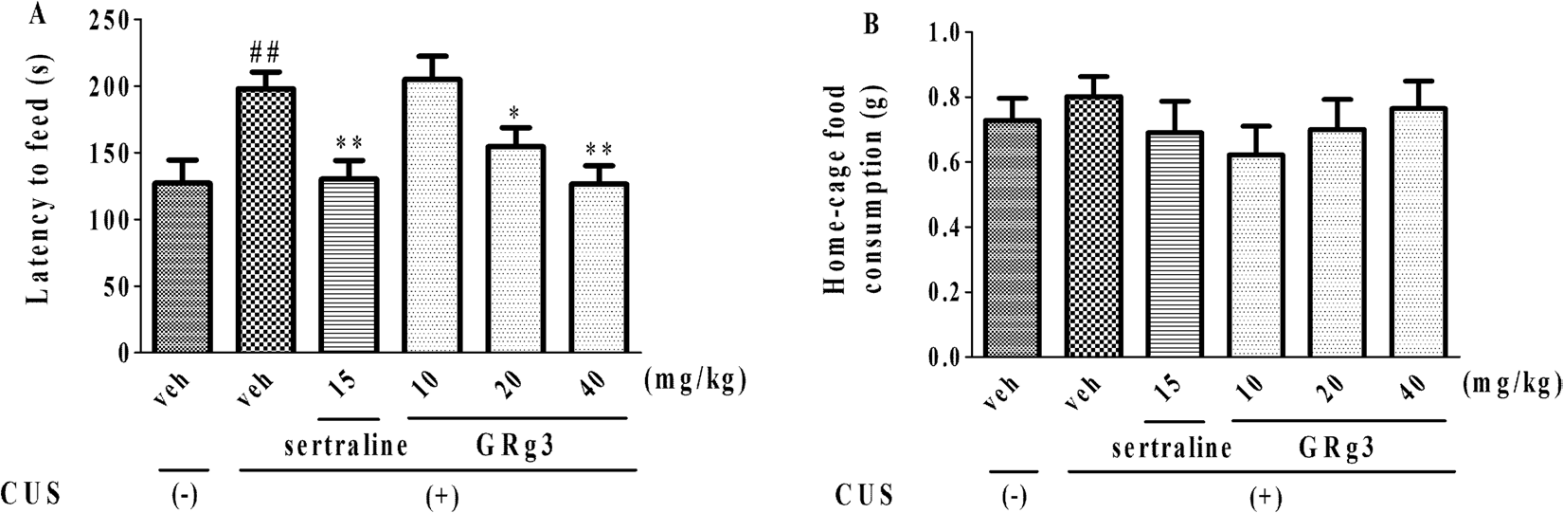
The anxiolytic-like effects of GRg3 in NSFT following exposure to CUS. The latency to feed was increased by CUS and reversed by GRg3. ^##^*p* < 0.01 vs. vehicle-treated CUS (-) group; *p < 0.05, **p < 0.01 vs. vehicle-treated CUS (+) group (n = 10).

As observed in Fig. 4, the latency to feed was increased following the CUS administration. Consistent with the results of sertraline (15 mg/kg, i.g), increased latency (F_5,54_ = 5.845, *p* < 0.05, 4 A) was antagonized by GRg3 (20 and 40 mg/kg, i.g). Moreover, no differences of in home-cage food consumption were obtained (F_5,54_ = 0.5692, *p* > 0.05, 4B) among groups, indicating that CUS-induced behavioral deficits were ameliorated by GRg3 via NSFT.

### The locomotor activity in the anxiolytic-like activities of GRg3

**Figure 5:**
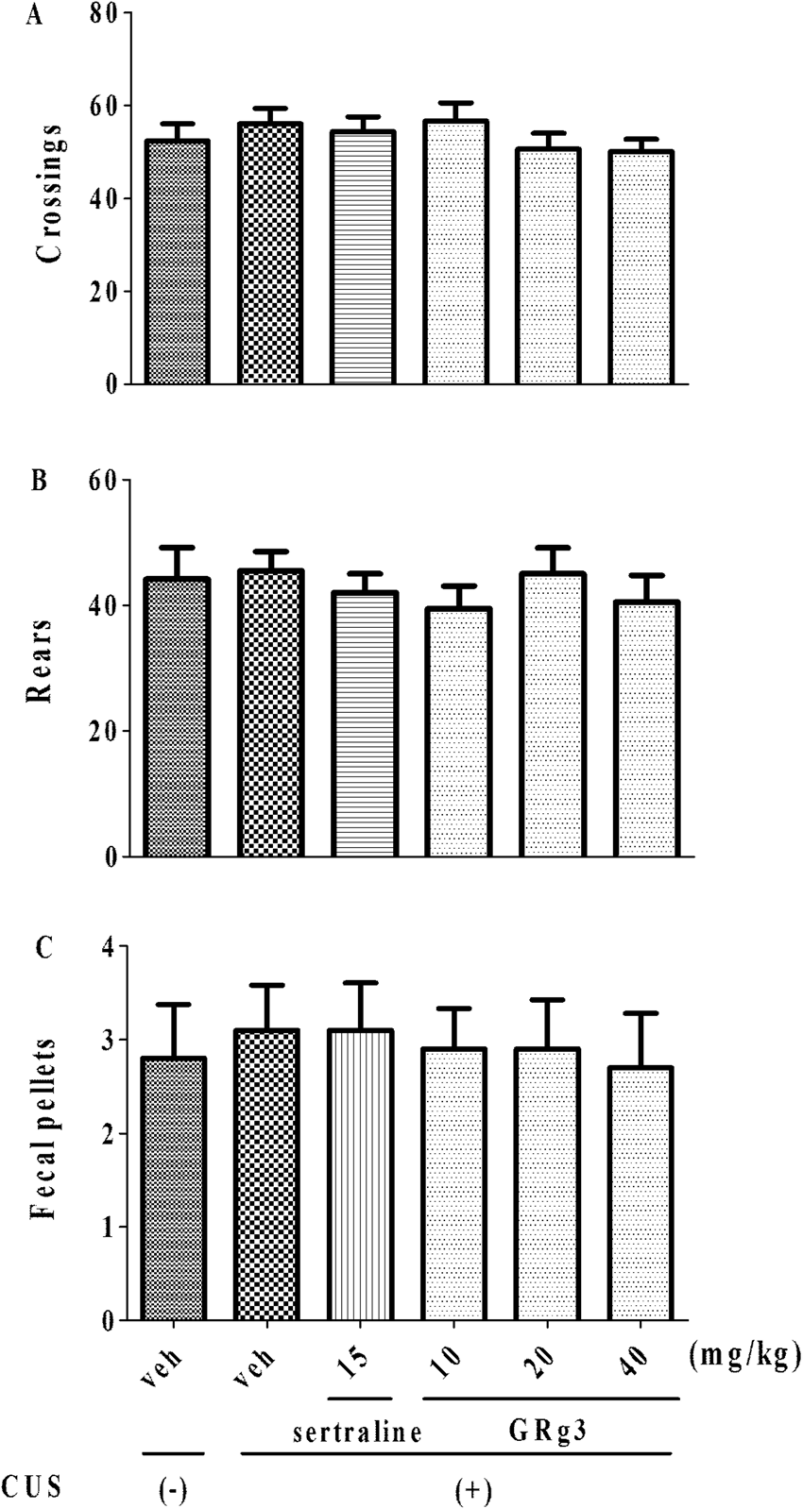
The effects of GRg3 on the locomotor activity. None of the treatments altered the number of line crossings (**A**), rears (**B**), and fecal pallets (**C**) in OFT (n = 10).

The impact of locomotor activity was shown in Fig. 5. No significant difference on crossings (F _5,54_ = 0.6847, *p* > 0.05, 5 A), rears (F_5,54_ = 0.4066, *p* > 0.05, 5B), and fecal pallets (F_5,54_ = 0.09539, *p* > 0.05, 5 C) was observed, suggesting that the anxiolytic-like effects were produced by GRg3 except affecting locomotion.

### The role of CUS-induced HPA axis changes in the effects of GRg3

**Figure 6:**
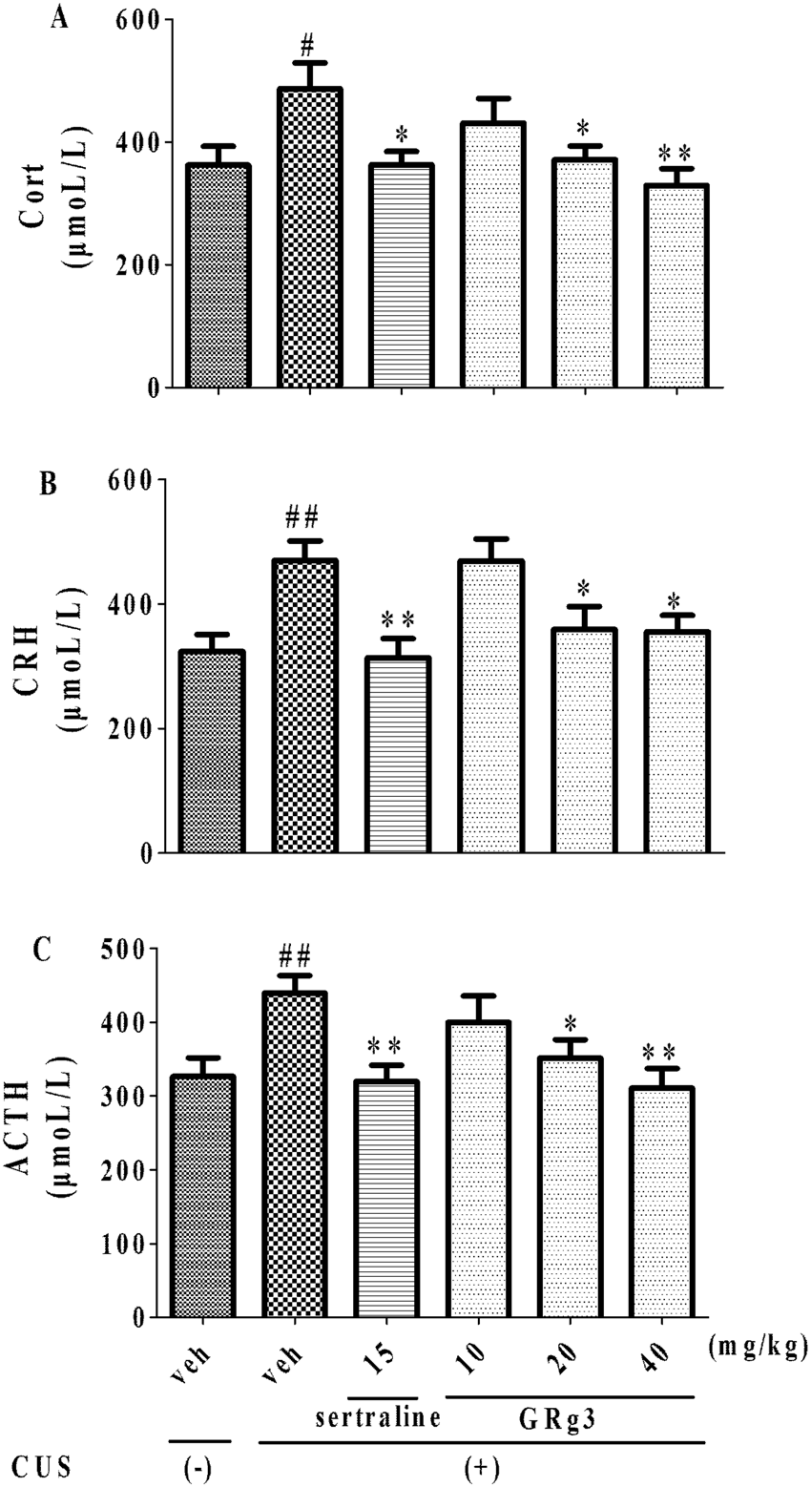
The effects of GRg3 on the levels of Cort (**A**), CRH (**B**), ACTH (**C**) in serum. ^#^*p* < 0.05, ^##^*p* < 0.01 vs. vehicle-treated CUS (−) group; *p < 0.05, **p < 0.01 vs. vehicle-treated CUS (+) group (n = 10).

The effects of GRg3 on levels of Cort, CRH and ACTH were shown in Fig. 6. Following the exposure to CUS, the levels of Cort (F_5,54_ = 3.356, *p* < 0.05, 6 A), CRH (F_5,54_ = 4.987, *p* < 0.05, 6B) as well as ACTH (F_5,54 = _3.658, *p* < 0.05, 6 C) in serum were obviously increased. In accordance with the effects of sertraline (15 mg/kg, i.g), elevated hormones above were also markedly blocked by GRg3 (20 and 40 mg/kg, i.g), respectively. The effects of induced by GRg3 were associated with decreased levels on Cort, CRH and ACTH.

### The role of neurosteroid levels in the anxiolytic-like effects of GRg3

**Figure 7:**
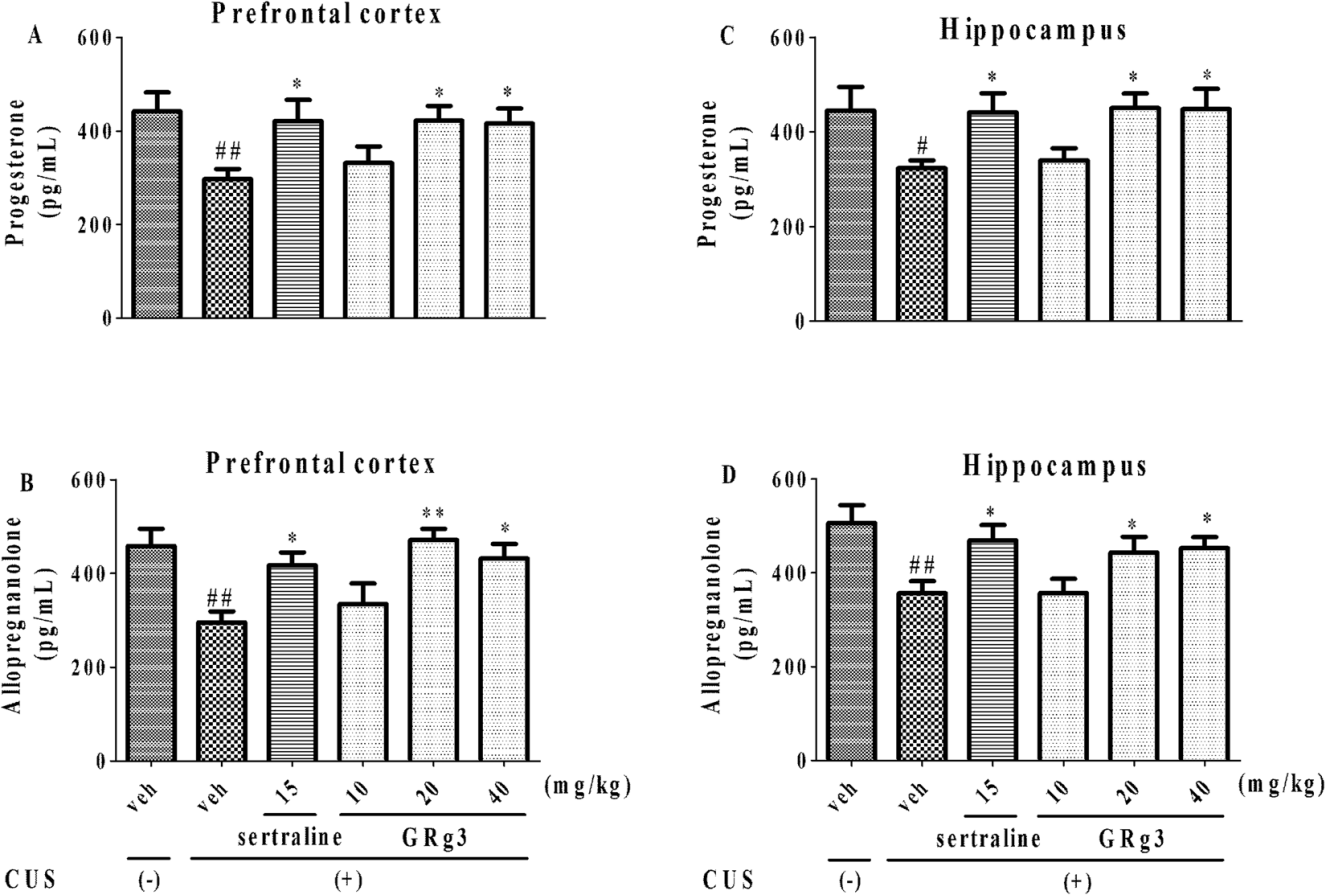
The effects of GRg3 on the levels of progesterone and allopregnanolone in the prefrontal cortex (**A**,**C**) and hippocampus (**B**,**D**), respectively. ^#^*p* < 0.05, ^##^*p* < 0.01 vs. vehicle-treated CUS (−) group; ^*^p < 0.05, ^**^p < 0.01 vs. vehicle-treated CUS (+) group (n = 10).

In Fig. 7, levels on progesterone and allopregnanolone in both regions were decreased after exposure to CUS, respectively. Like sertraline (15 mg/kg, i.g.), both decreased levels on neurosteroids were reversed by GRg3 (20 and 40 mg/kg, i.g.) in the prefrontal cortex (F_5,54_ = 2.805, *p* < 0.05, for progesterone, 7 A; F_5,54_ = 4.897, *p* < 0.05, for allopregnanolone, 7B) and hippocampus (F_5,54_ = 2.716, *p* < 0.05, for progesterone, 7 C; F_5,54_ = 3.973, *p* < 0.05, for allopregnanolone, 7D), respectively. Thus, anxioytic-like effects of GRg3 were relevant to biosynthesis of progesterone and allopregnanolone in the brain.

### The levels on monoamines in anxiolytic-like effects of GRg3

The effects of GRg3 on levels of monoamines in the brain were observed in Tables 2 and 3. After the exposure to CUS, the levels on 5-HT in both regions were decreased, respectively. Similar to the effects of sertraline (15 mg/kg, i.g.), decreased levels on 5-HT (F_5,54_ = 2.435, *p* < 0.05, for prefrontal cortex, Table 2; F_5,54_ = 2.457, *p* < 0.05, for hippocampus, Table 3) were blocked by GRg3 (20 and 40 mg/kg, i.g.), respectively.

**Table 2:** The effects of GRg3 on prefrontal cortex monoamine neurotransmitter levels in CUS rats.

Groups	5-HT	5-HIAA	NE	AD	HVA	DA	DOPAC
CUS (-)	253.1 ± 40.42	188.4 ± 24.68	188.0 ± 13.42	184.5 ± 17.68	142.2 ± 13.86	147.0 ± 18.16	206.5 ± 23.06
CUS (+)	151.7 ± 14.82^#^	188.8 ± 26.51	200.2 ± 23.55	151.7 ± 21.62	149.3 ± 9.957	149.9 ± 14.94	190.3 ± 24.56
Sertraline 15 mg/kg	268.1 ± 27.59*	208.4 ± 33.00	203.5 ± 29.70	177.9 ± 22.76	165.6 ± 12.20	142.1 ± 12.87	197.8 ± 25.45
GRg3 10 mg/kg	190.6 ± 28.86	206.5 ± 29.03	195.6 ± 26.19	163.4 ± 15.03	167.7 ± 21.79	142.9 ± 20.71	208.4 ± 24.80
GRg3 20 mg/kg	265.2 ± 41.50*	206.3 ± 26.94	190.9 ± 25.84	162.2 ± 24.78	139.1 ± 10.56	172.4 ± 19.20	209.3 ± 25.96
GRg3 40 mg/kg	271.1 ± 31.73*	190.1 ± 22.84	181.9 ± 19.25	149.2 ± 18.90	142.4 ± 9.398	172.3 ± 19.20	194.9 ± 24.60

^#^*p* < 0.05 vs. vehicle-treated CUS (−) group; ^*^p < 0.05 vs. vehicle-treated CUS (+) group (n = 10).

**Table 3:** The effects of GRg3 on hippocampal monoamine neurotransmitter levels in CUS rats.

Groups	5-HT	5-HIAA	NE	AD	HVA	DA	DOPAC
CUS (−)	293.3 ± 21.27	249.9 ± 29.42	215.1 ± 13.50	192.3 ± 26.36	162.2 ± 20.17	207.9 ± 25.06	181.3 ± 27.50
CUS (+)	206.1 ± 21.32^#^	217.1 ± 24.31	211.6 ± 27.84	180.7 ± 29.97	178.9 ± 27.96	191.7 ± 23.64	211.6 ± 34.22
Sertraline 15 mg/kg	289.5 ± 45.02*	212.1 ± 22.04	245.0 ± 33.79	167.6 ± 27.60	160.1 ± 22.62	199.3 ± 22.80	209.4 ± 34.63
GRg3 10 mg/kg	203.8 ± 24.48	214.8 ± 32.00	195.9 ± 32.44	172.3 ± 25.52	168.7 ± 29.67	234.9 ± 35.46	214.5 ± 34.78
GRg3 20 mg/kg	308.3 ± 33.98*	204.2 ± 18.98	203.8 ± 30.29	198.9 ± 29.40	200.3 ± 27.23	224.3 ± 32.72	187.7 ± 22.56
GRg3 40 mg/kg	279.4 ± 23.03*	199.8 ± 29.85	247.2 ± 25.08	164.8 ± 20.67	170.8 ± 24.09	220.4 ± 27.55	225.4 ± 29.39

^#^*p* < 0.05 vs. vehicle-treated CUS (−) group; ^*^p < 0.05 vs. vehicle-treated CUS (+) group (n = 10).

However, AD (F_5,54_ = 0.4730, *p* > 0.05, for prefrontal cortex, Table 2; F_5,54_ = 0.2656, *p* > 0.05, for hippocampus, Table 3), 5-HIAA (F_5,54_ = 0.1305, *p* > 0.05, for prefrontal cortex, Table 2; F_5,54_ = 0.4462, *p* > 0.05, for hippocampus, Table 3), DA (F_5,54_ = 0.6384, *p* > 0.05, for prefrontal cortex, Table 2; F_5,54_ = 0.3328, *p* > 0.05, for hippocampus, Table 3), NE (F_5,54_ = 0.1152, *p* > 0.05, for prefrontal cortex, Table 2; F_5,54_ = 0.4986, *p* > 0.7748, for hippocampus, Table 3), HVA (F_5,54_ = 0.8480, *p* > 0.05, for prefrontal cortex, Table 2; F_5,54_ = 0.3336, *p* > 0.05, for hippocampus, Table 3), DOPAC (F_5,54_ = 0.1030, *p* > 0.05, for prefrontal cortex, Table 2; F_5,54_ = 0.3007, *p* > 0.05, for hippocampus, Table 3) were not significantly affected by GRg3. Accordingly, anxiolytic-like effects of GRg3 were involved with the normalized levels on 5-HT in both regions.

## Discussion

The anxioytic-like activities of GRg3 were preliminarily evaluated. The anxioytic effects were produced by GRg3 except affecting the locomotion. Moreover, based on results of neurosteroids biosynthesis, monoamine neurotransmitters and hormones of HPA axis, the anxioytic-like effects of GRg3 were involved in normalization of HPA axis dysfunction, biosynthesis of neurosteroids and serotonergic system.

Anxiety is one of the serious mental disorders in the world^28^. CUS induces behavioral deficits that resemble the anxiogenic-like behavior^20,25^. The CUS model is similar to the anxiogenic-like symptoms and widely selected in the anxiolytic evaluation^20^. NSFT and EPMT are used to evaluate the anxiolytic effects, and also sensitive to anxiolytic treatments^5,25^. The present study showed that the increased latency to feed in NSFT and the decreased time/entries of open arms in EPMT, two indicators of the anxiogenic-like symptoms, were induced by CUS.

The CUS-induced behavioral deficits could be blocked by the repeated administration of anxiolytic treatments^25^. In line with the effects of sertraline (15 mg/kg i.g.), the increased latency to feed was reversed by GRg3 (20 and 40 mg/kg i.g.) except affecting home-cage food consumption in NSFT. In addition, the decreased time/entries in open arms were also antagonized by GRg3 at the same doses except affecting the total time/entries in EPMT. The effective doses of GRg3 (20 and 40 mg/kg i.g.) were confirmed between NSFT and EPMT and in line with its antidepressant-like effects^15^. Moreover, consistent with the previous findings^29^, the locomotor activity was not affected by GRg3, which were also consistent with total time and entries in EPMT. Based on the previous and presents studies, the anxiolytic-like effects were produced by GRg3 except affecting locomotor activity.

Dysfunction in prefrontal cortex or hippocampus is implicated in the pathogenesis of anxiogenic-like behavioral deficits^5^. Both brain regions are involved in explicit memory, fear conditioning and emotional processing. To investigate the significance of neurosteroids in the anxiogenic-like effects of GRg3, levels on neurosteroids and monoamine neurotransmitters were also assessed.

The involved factors of anxiogenic-like behavior are not known clearly. More evidences demonstrate that dysfunction of neurosteroids biosynthesis (e.g. progesterone and allopregnanolone) is considered as one of the possible factors to anxiety^19^. Like sertraline (15 mg/kg i.g.), both decreased neurosteroids were significantly reversed by GRg3 in prefrontal cortex and hippocampus, respectively. Anxiolytic-like effects of GRg3 on CUS-induced behavioral deficits may be associated with the biosynthesis of progesterone and allopregnanolone in the brain. Consistently, the altered levels of progesterone affected the metabolite steroid (i.e allopregnanolone). Decreased the levels of allopregnanolone in the brain were dramatically induced by progesterone withdrawals^30^.

Progesterone is thought to be one of the important precursor molecule for 3β-pregnane neuroactive steroids that regulate the anxiolytic-like activities^10,19^. The positve effects of progesterone may produce following its conversion to allopregnanolone that metabolite’s agonistic acts on GABA (γ-aminobutyric acid) A receptors^19,31^. The GABAA agonist modulator interacted by regulating the expression of GABAA receptor subunits to produce the neuroprotective effects^19^. Conversely, the anxiogenic-like behavior is closely relevant to dysfunction of neurosteroids biosynthesis. For instance, the decreased levels on allopregnanolone in peripheral blood or cerebrospinal fluid (CSF) are associated wih anxiety, premenstrual dysphoric disorders, schizophrenia, or/and impulsive aggression^32^.

Besides neurosteroids biosynthesis, the hyperactivity of the HPA axis, is commonly observed in patients with anxiety^33^. Here, the increased levels on Cort, CRH and ACTH following CUS were shown. The results were partially supported by that the elevated levels on CRH, Cort and ACTH in depressive- or anxiogenic- like behvioral deficits in rodents^34,35^. Unanimously, allopregnanolone is considered as one of the endogenous negative regulators in HPA axis activity. Cort was elevated concomitantly with decreased levels on allopregnanolone after exposure to CUS^36^. Interestingly, the stress hormones of HPA axis above in post-CUS rats could be blocked by GRg3, suggesting that the normalization of neurosteroid levels and HPA axis dysfunction may be associated with anxiolytic-like activities of GRg3.

Moreover, monoaminergic system closely interacts in central nervous system (CNS) (particularly in prefrontal cortex and hippocampus) and is involved in anxiogenic disorders. Accordingly, the effects of monoamines in the anxiolytic-like effects of GRg3 were also evaluated. After exposure to CUS, the levels on 5-HT in prefrontal cortex and hippocampus were decreased that was in line with the previous observation^24^. In addition, monoaminergic hypothesis indicates that lowered levels on 5-HT in CNS are closely associated with the anxiogenic-like behavior^5^. However, similar to the effects of sertraline, the decreased levels on 5-HT were significantly blocked by GRg3, suggesting that anxioytic-like effects of GRg3 were also associated with normalization of levels on 5-HT.

Our findings were also in line with that GRg3 could reduce or partially antagonize the neurotoxic effects induced by Acrylamide towards the normal values of controls, including 5-HT, Cort, progesterone, estradiol, *et al*.^37^. Moreover, the antidepressant-like effects of GRg3 were at least partially associated with normalization of the dysfunction on 5-HT in brain^15^. In addition, although no reports show the effects of GRg3 on the HPA stress hormones, other ginsenoside active component (e.g GRg1) allviates PTSD-like behavioral deficits by reducing the Cort and CRH levels^38^. Thus, it seems that Grg3 may be causal in the observed changes in stress hormone levels in HPA axis, neurosteroids biosynthesis, and monoamine neurotransmitters. In addition, the observed changes in these indices may be a chain of events leading to the observed read outs. For instance, the neurosteriods biosyntheis may be considered as one of endogenous negative regulators of HPA axis activity^36^. Moreover, the study on HPA axis activity and in 5-HT system provides evidences to suggest that 5-HT system has a higher potential for stimulating the HPA axis. It supports that a stimulatory influence of 5-HT on HPA axis in humans and rodents is partially mediated by 5-HT 1 A receptor subtype^39^. Futhermore, reduced neurosteroids (i.e allopregnanolone and pregnanolone) are potential neuromodulators able to affect a number of membrane receptors, including GABA, N-methyl-D-aspartate (NMDA), 5-HT, *et al*.^40^.

Summary, GRg3 produces the anxioytic-like activities that may be associated with biosynthesis of neurosteroids, normalization of serotonergic system and HPA axis abnormality, which may account for pathology underlying anxioytic-like effects of GRg3. Accordingly, the results not only promote our knowledge in anxiety, but also provide clinical implications for GRg3 that maybe considered as a novel drug for anxiety. Although anxioytic-like effects of GRg3 are preliminarily evaluated, many relevant molecular readouts are not fully found out. Further researches should be conducted molecular pathways/targets and pharmacodynamics on anxioytic-like effects of GRg3.
